# The established and emerging roles of astrocytes and microglia in amyotrophic lateral sclerosis and frontotemporal dementia

**DOI:** 10.3389/fncel.2015.00414

**Published:** 2015-10-27

**Authors:** Rowan A. Radford, Marco Morsch, Stephanie L. Rayner, Nicholas J. Cole, Dean L. Pountney, Roger S. Chung

**Affiliations:** ^1^Department of Biomedical Sciences, Faculty of Medicine and Health Sciences, Macquarie UniversitySydney, NSW, Australia; ^2^Menzies Health Institute Queensland, Griffith UniversityGold Coast, QLD, Australia

**Keywords:** amyotrophic lateral sclerosis, frontotemporal dementia, astrocyte, microglia, neuroinflammation, phagocytosis, glymphatic, vasculature

## Abstract

Amyotrophic lateral sclerosis (ALS) and frontotemporal dementia (FTD) are two progressive, fatal neurodegenerative syndromes with considerable clinical, genetic and pathological overlap. Clinical symptoms of FTD can be seen in ALS patients and *vice versa*. Recent genetic discoveries conclusively link the two diseases, and several common molecular players have been identified (*TDP-43, FUS, C9ORF72*). The definitive etiologies of ALS and FTD are currently unknown and both disorders lack a cure. Glia, specifically astrocytes and microglia are heavily implicated in the onset and progression of neurodegeneration witnessed in ALS and FTD. In this review, we summarize the current understanding of the role of microglia and astrocytes involved in ALS and FTD, highlighting their recent implications in neuroinflammation, alterations in waste clearance involving phagocytosis and the newly described glymphatic system, and vascular abnormalities. Elucidating the precise mechanisms of how astrocytes and microglia are involved in ALS and FTD will be crucial in characterizing these two disorders and may represent more effective interventions for disease progression and treatment options in the future.

## Introduction

**Figure 1:**
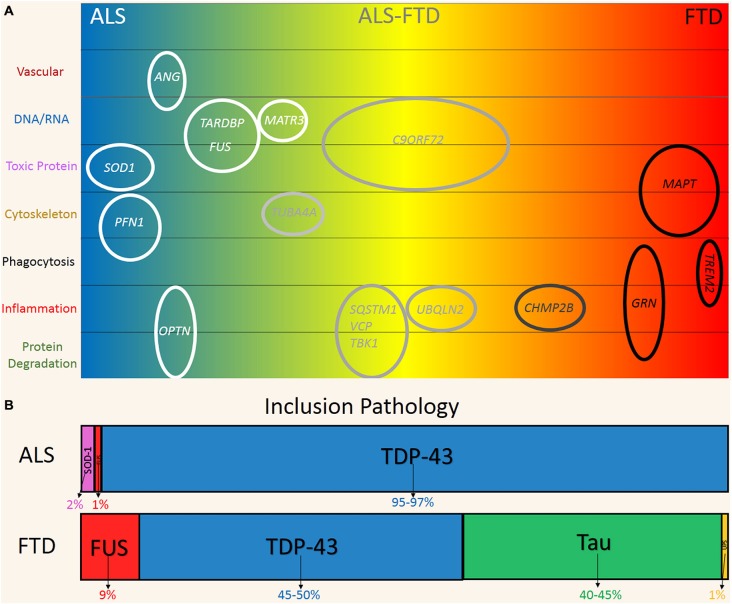
**Genetic and pathological overlap between amyotrophic lateral sclerosis (ALS) and frontotemporal dementia (FTD). (A)** Familial and sporadic genetic mutations were linked to the clinical phenotypes on the ALS (blue) and FTD (red) spectrum. Genes are plotted according to their hypothesized mechanism in relation to disease (top to bottom). **(B)** Pathological protein inclusions are a hallmark of ALS and FTD, reflecting the significant overlap on the disease spectrum. FUS (Red) and TDP-43 (Blue) inclusions are found in both ALS and FTD. Predominate SOD1 (Pink) and Tau (Green) is more indicative of ALS and FTD respectively. FTD-UPS (Yellow) is found in ~1% of cases and represent cases of familial *CHMP2B* mutations.

Amyotrophic lateral sclerosis (ALS) is a fatal and rapidly progressing multisystem neurodegenerative syndrome, characterized by the degeneration of the motor neurons (MNs) in the motor cortex, brainstem and spinal cord (Hardiman et al., 2011). Symptoms present first as focal upper and/or lower MN dysfunction of a skeletal muscle group which progressively deteriorates, ultimately spreading to other muscle groups (Ravits, 2014). Disease progression is rapid, with 50% of patients dying due to respiratory complications within 3 years of symptom onset (Kiernan et al., 2011). Over the last 25 years, it has become increasingly apparent that ALS shares significant overlap with another progressive and fatal neurodegenerative syndrome Frontotemporal dementia (FTD). Up to 50% of patients with ALS develop FTD symptoms and approximately 15% of FTD patients display MN dysfunction typical of ALS (Ng et al., 2015). Besides this clinical connection, ALS and FTD also share significant genetic and pathological overlap (Bennion Callister and Pickering-Brown, 2014), represented in Figure 1. However, the causal mechanism/s of both syndromes are currently unknown and treatment is largely symptomatic (Hardiman et al., 2011; Piguet et al., 2011).

Five years ago, large hexanucleotide repeat expansions (~100–1600 G_4_C_2_ repeats) of intronic regions of the *C9ORF72* gene were discovered in sporadic and familial forms of ALS and FTD (Renton et al., 2014). These studies provided seminal evidence for a direct molecular link between these two conditions. The repeat expansions are now recognized as the most common known mutation in both familial and sporadic ALS and FTD. Expansions have been identified in up to 40% and 25% of familial cases and ~6% and 7% of sporadic or seemingly non-inherited forms of ALS and FTD respectively (Robberecht and Philips, 2013; Renton et al., 2014). Multiple other genes have also been linked to ALS and FTD. Mutations in the genes *TARDBP* and fused in sarcoma (*FUS*), which respectively encode for the proteins TDP-43 and FUS, are associated with ~9% of familial, 2% of sporadic cases of ALS and rarely in FTD (Renton et al., 2014). The *MAPT* gene encodes microtubule associated protein tau (tau) and mutations have been identified in ~2–11% of familial FTD cases (Sieben et al., 2012). *SOD1* is another gene that is strongly associated with ALS with mutations found in 12–20% of familial and 1–2% of sporadic cases (Al-Chalabi et al., 2012). With the exception of *MAPT* these genes are not segregated to neurons and are expressed by glia and various other cell types, which suggests a multicellular pathogenesis.

The presence of ubiquitinated, cytoplasmic inclusions in neurons and some glia is a pathological hallmark shared by the two disorders (Ng et al., 2015). Figure 1B shows the distribution of inclusion pathology seen in both ALS and FTD. In ~95% of ALS and 50% of FTD cases, these inclusions are predominately comprised of TDP-43. FUS protein inclusions are found in ~1% and 10% of ALS and FTD cases respectively (Mackenzie et al., 2010). Tau inclusion pathology is more characteristic of FTD (~40% of cases) and is only found rarely in cases of ALS (Dickson et al., 2011; Ng et al., 2015). At the other end of the spectrum, SOD-1 inclusion pathology is seen in ~2% of ALS cases and is incredibly rare in FTD with only one case reported (Bennion Callister and Pickering-Brown, 2014). Taken together, the pathogenic and genetic features represent a clear commonality between ALS and FTD, which are now believed to exist on a phenotypic continuum (Ling et al., 2013).

In addition to protein inclusions, another feature of ALS and FTD neuropathology is reactive gliosis, which is characterized by astrocytic hypertrophy and microglial proliferation (Al-Chalabi et al., 2012; Ng et al., 2015). Reactive gliosis is an indicator of neuroinflammation (Streit et al., 2004) and occurs in areas of neuronal loss and inclusion pathology in ALS and FTD (Brettschneider et al., 2012). Studies over the past 15 years have strongly indicated that ALS and FTD propagate via multiple cell types, with reactive gliosis being heavily implicated (Ilieva et al., 2009). Astrocytes and microglia in particular have been shown to be associated with disease progression and spreading (Philips and Robberecht, 2011).

Various pathways have been implicated to contribute to ALS and FTD neurodegeneration, including inflammation, RNA toxicity and altered splicing/expression (DNA/RNA homeostasis), and cytoskeletal, vascular and protein dysfunction (Lagier-Tourenne and Cleveland, 2009; Garbuzova-Davis et al., 2012; Ravits et al., 2013). Both microglia and astrocytes can be compromised through a variety of these signaling pathways that result in deregulated glia–motor neuron communication. However, the precise contribution of glial cells and their exact involvement in ALS and FTD pathology is currently under intense investigation. Here we aim to summarize the established and novel implications of astrocyte and microglia in ALS and FTD, identifying key aspects of the neuroinflammatory involvement, microglia phagocytosis, defective waste clearance and circulatory dysfunction.

## The Pathogenic Role of Neuroinflammatory Glia in ALS

Inflammatory glia have been repeatedly reported in animal models of FTD (see Roberson, 2012). Yet, current models of tau pathology describe clinically a very heterogeneous group, including FTD and Parkinsonism, and our focus in this review will therefore be on the neuropathological characterization of inflammatory glia in ALS. Significant insight into the pathogenic role of glia in disease progression has been revealed through allografted chimeric and conditional knock out studies using ALS mutant SOD-1 rodent models (see Robberecht and Philips, 2013). These studies have convincingly shown that both astrocytes and microglia/myeloid progenitors significantly influence the progression of neurodegeneration in these models. More recent studies have added weight to the existing evidence that astrocytes contribute to ALS progression by utilizing mice xenografted with human glial progenitors generated from induced pluripotent stem cells (iPSCs). Grafted glial progenitors from patient iPSCs with familial *SOD1* mutations differentiated into astrocytes and induced MN degeneration and motor deficits in WT mice. Progenitors from healthy individuals without ALS linked mutations did not contribute to an ALS phenotype (Chen et al., 2015). In another study, iPSC derived glial progenitors from healthy individuals (lacking ALS mutations) formed astrocytes that increased the survival of MNs when transplanted at disease onset in a mutant SOD-1 mouse model (Kondo et al., 2014). Such *in vivo* studies provide important insight into the pathogenic role of ALS patient glia and demonstrate a potential mechanism of how glia can influence the progression of neurodegeneration (i.e., modifying the molecular phenotype and function).

Notably, astrocytes retrieved from post-mortem central nervous system (CNS) of familial (*SOD1* and unidentified) and sporadic cases were also neurotoxic to co-cultured MNs (Haidet-Phillips et al., 2011; Re et al., 2014). While the necrotic environment of post-mortem tissue has to be considered, another study reported that astrocytes generated from sporadic and familial (*C9ORF72* and *SOD1*) ALS iPSCs were also toxic to co-cultured MNs (Meyer et al., 2014). These findings correlate with studies that report the neurotoxicity of glia derived from SOD-1 transgenic mice (Di Giorgio et al., 2007; Nagai et al., 2007). Collectively, this glia-induced neurotoxicity suggests a common mechanism in both sporadic and familial ALS.

Different experimental approaches further suggest that astrocytes are neurotoxic to MNs in the context of ALS. A recently reported ubiquitous RNAi knockdown of TDP-43 in mice led to severe neurodegeneration and an ALS phenotype. The study revealed a greater knockdown of TDP-43 in astrocytes compared to MNs, significant astrogliosis and marked upregulation of lipocalin-2 expression in reactive astrocytes (Yang et al., 2014). Lipocalin-2 is a feature of inflammatory astrocytes (Zamanian et al., 2012) and can enhance reactive astrogliosis via autocrine signaling (Lee et al., 2009). Specific knockout of TDP-43 in cortical and MNs in mice produced a less severe phenotype, which further highlights the contribution of multiple cell types in ALS and FTD (Wu et al., 2012b; Iguchi et al., 2013).

Interestingly, overexpression of ALS associated mutant TDP-43 driven by an astrocytic promoter was sufficient to cause MN degeneration in rats and was also associated with marked up-regulation of lipocalin-2 in reactive astrocytes (Tong et al., 2013). Overexpression of mutant TDP-43 in rat neurons also induced gliosis and lipocalin-2 upregulation in surrounding reactive astrocytes. The analysis of post-mortem frontal cortex of FTD patients likewise revealed an increase in lipocalin-2, and that recombinant lipocalin-2 was exclusively toxic to cultured neurons (Bi et al., 2013). Huang et al. (2014) reported that inducible overexpression of mutant TDP-43 can increase lipocalin-2 expression in cultured astrocytes and while pathological mutations can lead to alterations in RNA homeostasis similar to those seen in knockdown studies. Further studies are clearly needed to understand the mechanism of pathological TDP-43 and the ambiguous role of lipocalin-2 in ALS and FTD pathogenesis and its potential as a therapeutic target or biomarker for assessing neuroinflammation.

Recent advances in patient neuroimaging have allowed direct visualization of neuroinflammation such as gliosis. Positron Emission Tomography (PET) and Single Positron Emission Computed Tomography (SPECT) or Magnetic Resonance Imaging (MRI) in patients targeting activated microglial receptors or astrocytic metabolites have shown gliosis throughout various symptomatic stages of ALS and FTD while absent in non-disease controls (Cagnin et al., 2004; Chiò et al., 2014). This, along with data from animal models, strongly indicates that gliosis is unlikely to represent a specific event only seen in post-mortem tissue at the end stage of ALS and FTD. More recently, modalities that specifically evaluate astrocyte metabolism (e.g., radiopharmaceutical acetate derivatives; Marik et al., 2009; Ouyang et al., 2014) could be used to monitor astrogliosis in patients more accurately. New imaging ligands such as the modern translocator protein ligands allow for tracking microglia activation with higher specificity and reduced radiation dosage (Corcia et al., 2012). Collectively, these rapidly improving technologies are revealing important information regarding the involvement of astrocytes and microglia in various stages of degeneration in ALS and FTD patients.

## The Role of Glial Phagocytosis in ALS and FTD

**Figure 2:**
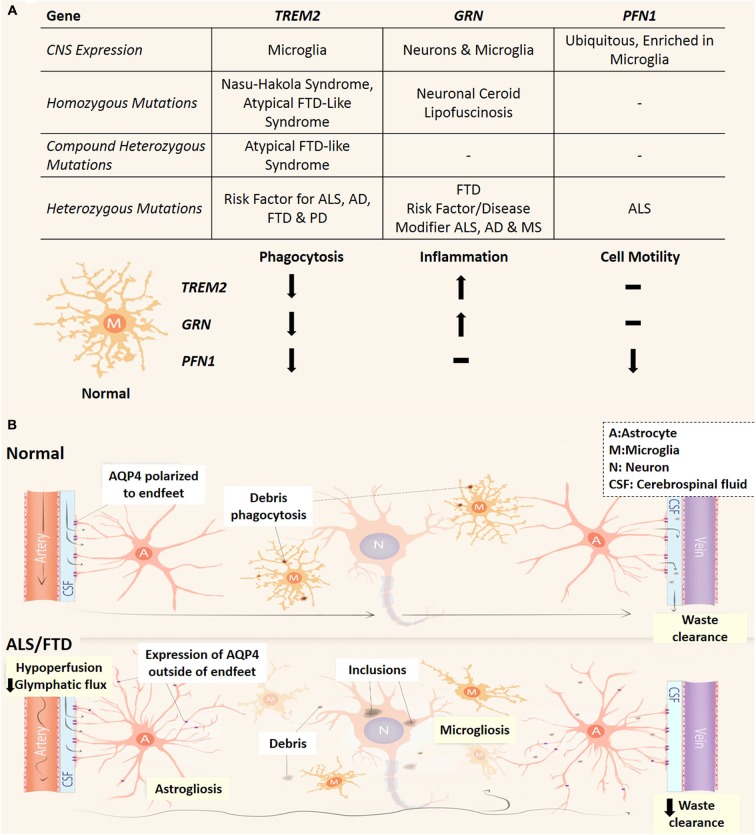
**Phagocytic dysfunction and the glymphatic pathway and its (potential) involvement in ALS and FTD. (A)** Three genes (*TREM2, GRN* and PFN1) which link microglial phagocytic dysfunction to ALS and FTD and their effect on microglial phenotype. All three are predicted loss of function mutations which decrease the phagocytic capacity of microglia. Depending on the type of mutation/s to these genes different neurodegenerative conditions can arise, while variants cause increase risk of developing neurodegenerative conditions or worsen prognosis. Neuronal Ceroid Lipofuscinosis is a type of neurodegenerative lysosomal disorder which has been reported in patients *PRGN* null patients (Petkau and Leavitt, 2014). AD, Alzheimer’s disease; PD, Parkinson’s disease; MS, multiple sclerosis. **(B)** In the normal CNS, CSF circulates in a perivascular compartment driven by arterial pulse pressure. Astrocytic endfeet cover the perivascular space and facilitate movement of CSF into the parenchyma largely via AQP4. This fluid flow through the interstitial space allows the removal of debris from the extracellular space before draining into venous perivascular compartments. Microglia also remove potentially toxic waste via phagocytosis and dysfunctional microglial phagocytosis is linked to ALS and FTD pathogenesis (see **A**). In the CNS of ALS and FTD patients, glymphatic function and microglial phagocytosis may be compromised and contribute to neurodegeneration. Reactive astrocytes conceivably lose AQP4 polarization and express it elsewhere. This may lead to turbulent flow through the interstitium. Cerebral vascular function is reduced in patients which could potentially lead to decreased pressure for glymphatic function. A, astrocytes; M, microglia; N, neuron; CSF, cerebrospinal fluid.

Astrocytes have been found to highly express an array of phagocytic receptors and actively contribute to this process by phagocytosing synapses and axonal mitochondria in the developing and adult CNS (Chung et al., 2013; Davis et al., 2014). Nonetheless, microglial cells have been shown to be the main culprit for phagocytosis and synaptic pruning that is crucial to CNS function by removing potentially toxic debris and the reorganization of the CNS connectome (Neumann et al., 2009; Xavier et al., 2014). The altered phagocytic activity of microglia has been implicated in multiple neurodegenerative disorders. This link has been highlighted through three discoveries of genetic mutations in phagocytosis-related genes in ALS and FTD patients (Figure 2A). *TREM2* is exclusively expressed by microglia in the CNS (Colonna, 2003; Thrash et al., 2009) and missense variants have been recognized as a risk factor for ALS, FTD, Alzheimer’s and Parkinson’s disease (Rayaprolu et al., 2013; Cady et al., 2014; Harms et al., 2014). Furthermore, recessive mutations in *TREM2* are also associated to an orphan neurodegenerative condition known as Nasu-Hakola Syndrome with patients presenting with lytic bone cysts, atypical FTD and psychiatric dysfunction (Kaneko et al., 2010). Interestingly homozygous and compound heterozygous mutations are linked to a familial FTD-like disorder without bone involvement (Kaneko et al., 2010; Guerreiro et al., 2013; Borroni et al., 2014). These mutations in *TREM2* are proposed to confer loss of TREM2 protein function, which causes decreased microglial phagocytosis and altered inflammatory responses (Kleinberger et al., 2014; Wang et al., 2015). Additionally, dysfunctional microglial phagocytosis is directly linked to FTD via mutations in *GRN* (progranulin) and may confer elevated risk of developing Alzheimer’s disease and ALS (Petkau and Leavitt, 2014). Progranulin is expressed by neurons and microglia and following secretion can act as a neuroinflammatory modulator (Petkau et al., 2010), and facilitate microglial recognition of apoptotic cells and potentially toxic elements such as amyloid beta (Aβ; Pickford et al., 2011; Minami et al., 2014). Also, loss of function mutations in *PFN1* (profilin 1) have been identified in familial ALS (Wu et al., 2012a) and profilin has been shown to be essential in regulating actin dynamics necessary for phagocytosis, phagosome formation and is upregulated in microglia following insult (Pearson et al., 2003; Dong et al., 2004; Kim et al., 2012). Research into how these *PFN1* mutations influence microglia function will be crucial to understanding the pathogenicity of those mutations. While progranulin and profilin 1 mutations are likely to impact multiple cell types (especially neurons), cell specific transcriptome analysis of the mouse cortex indicates that all three genes are highly transcribed in microglia (Zhang et al., 2014). Taken together, these studies highlight a potential link between reduced microglial phagocytic capacity and the development of neurodegeneration, ALS and particularly FTD and is represented in Figure 2A.

Phagocytosis is incomplete without the intracellular breakdown of engulfed material. Autophagy is an essential component of this internal degradation inside phagocytes as autophagosome-lysosome fusion is crucial to break down this debris. Alterations to this pathway are directly implicated in ALS, ALS-FTD, FTD and/or Multisystem Proteinopathy pathogenesis through mutations to *OPTN*, *SQST1*, *VCP* and the recently discovered *TBK1* (Renton et al., 2014; Freischmidt et al., 2015). These genes are vital to autophagosome formation, maturation and therefore crucial in LC3-assisted phagocytosis and intracellular waste clearance (Tresse et al., 2010; Seto et al., 2013). Interestingly these genes are also involved in inflammation as they can regulate Nuclear Factor-kappaB (NF-κB) signaling (Pomerantz and Baltimore, 1999; Asai et al., 2002; Zhu et al., 2007; Duran et al., 2008; Tresse et al., 2010; Seto et al., 2013). NF-κB is one of the major regulators of neuroinflammatory activation of glia (Zamanian et al., 2012) and its induction is seen in post-mortem ALS tissue, mutant SOD-1 and TDP-43 models (Migheli et al., 1997; Swarup et al., 2011a,b; Frakes et al., 2014). Any defects to these genes would potentially impact upon the function of innate immune cells (Deretic et al., 2013), particularly those segregated in the CNS like microglia and astrocytes. Determining how these genes are expressed, regulated and function in astrocytes and microglia will provide important insights into the neurodegenerative mechanisms underlying ALS and FTD.

## The Emerging Role of the Glymphatic System and Vascular Function in ALS and FTD

While phagocytosis of apoptotic neurons and cellular debris is a major pathway for removal of toxic substances within the CNS, the glymphatic system has recently emerged as a different clearance pathway with important immune functions (Iliff et al., 2012). The glymphatic system mediates circulation of cerebrospinal fluid (CSF) and exchange of interstitial fluid to remove extracellular waste (such as Aβ and tau proteins) and distribute compounds such as glucose, lipids, and neuromodulators to the CNS (Thrane et al., 2013; Xie et al., 2013; Iliff et al., 2014). The glymphatics run parallel to the CNS vasculature in a paravascular space enclosed by astrocytic endfeet (Figure 2B). Accordingly, the glymphatic system (reviewed in Jessen et al., 2015) relies heavily upon the vasculature in order to function as pressure differentials between arteries and veins propel the CSF through the CNS parenchyma (Iliff et al., 2013). The bulk of glymphatic flow through the CNS is facilitated by aquaporin-4 (AQP4), a water transporter specific to astrocytes in the CNS and polarized to their endfeet (Iliff et al., 2012; Papadopoulos and Verkman, 2013). Interestingly, elevated AQP4 with loss of astrocytic endfeet depolarization has been reported in transgenic mutant SOD-1 rat models and reactive astrocytes have been shown to up-regulate AQP4 elsewhere in the astrocytic arbor apart from the endfeet (Bataveljić et al., 2012; Papadopoulos and Verkman, 2013). Glymphatic flow significantly increased during non-rapid eye movement sleep and was largely controlled by norepinephrine (which modulates arousal) acting upon astrocytic α-adrenoceptors (Xie et al., 2013; Paukert et al., 2014). Increased levels of norepinephrine have been observed in the CSF, plasma and spinal cord tissue of ALS patients (Brooks et al., 1980; Bertel et al., 1991) and norepinephrine CSF levels were positively correlated with the severity of dementia in FTD (Engelborghs et al., 2008). It has been hypothesized that increased CSF levels of norepinephrine could lead to decreased glymphatic function, while any aberrant expression of AQP4 could potentially create turbulent convective flux through the CNS interstitium, ultimately leading to decreased removal of neurotoxic metabolites (Kress et al., 2014; Jessen et al., 2015). MRI imaging techniques allow live-imaging of the glymphatic system (Iliff et al., 2013; Yang et al., 2013) and are a novel approach to detect flow abnormalities in the glymphatic system in ALS and FTD patients. Recent studies have identified lymphatic vessels present in the dura mater, which drains CNS interstitial fluid via the glymphatic system and CSF from the subarachnoid space (Aspelund et al., 2015; Louveau et al., 2015). As T cells are implicated in the progression of ALS patients and animal models (Philips and Robberecht, 2011) this would reflect a novel way for lymphocytes to monitor and interact with CNS tissue via the glymphatic system and potentially influence neuroinflammatory events in ALS and FTD.

Glymphatic function is intimately linked to vascular flow via the parallel anatomy and requirement of pressure differentials created by blood flow. During development and in the mature CNS, astrocytes and microglia are crucial to complex signaling cascades and angiogenesis necessary for cerebrovascular function (see Abbott et al., 2006). Interestingly, two genes involved in vascular function have been linked to ALS. Mutations in *ANG* (angiogenin) have been found to segregate with both familial and sporadic forms of ALS and Parkinson’s disease (Greenway et al., 2006; van Es et al., 2011). Angiogenin was enriched and secreted by MNs with paracrine effects exclusively on astrocytes *in vitro* (Skorupa et al., 2012, 2013). *VEGFa* promoter haplotypes causing decreased expression also infer a greater risk of ALS. VEGFa is predominately expressed by astrocytes in the CNS (Zhang et al., 2014) and decreased VEGFa levels significantly reduce survival in mutant SOD-1 mice (Lambrechts et al., 2003). While decreased expression is a greater risk for ALS and can cause MN degeneration due to reduced ischemic tolerance (Oosthuyse et al., 2001), increased VEGFa expression by reactive astrocytes due to NF-κB-dependent pathways leads to greater infiltration of peripheral immune cells and blood brain barrier (BBB) breakdown in multiple sclerosis (MS) mouse models (Argaw et al., 2012; Chapouly et al., 2015). This highlights a potential dual role for VEGFa in neurodegeneration and how inappropriate control of VEGFa expression is associated with various forms of neurodegeneration. Further implicating the involvement of vascular defects, compromised blood brain and spinal cord barriers have been observed in post-mortem ALS and FTD tissue (De Reuck et al., 2012; Garbuzova-Davis et al., 2012). Early dysfunction of the blood spinal cord barrier has been shown to contribute to early MN damage in transgenic ALS-mutant SOD-1 mice (Zhong et al., 2008; Winkler et al., 2014). Further, patient cerebral perfusion neuroimaging studies have noticed hypoperfusion abnormalities in areas that correlate with neurodegeneration in ALS and FTD (Martin et al., 2001; Du et al., 2006; Zhong et al., 2008; Chiò et al., 2014; Winkler et al., 2014). Altogether these studies implicate vascular dysfunction in the pathogenesis of ALS and FTD.

## Conclusion

Research over the last decades has established that astrocytes and microglia play crucial roles in the development and/or progression of ALS and FTD through their complex interactions. Recent advances in iPSC technology have highlighted that glia secrete toxic factors that can trigger neurodegeneration. New gene discoveries have implicated that defects in glial phagocytic and neuroinflammatory activity are associated with neurodegeneration. There is now emerging evidence suggesting that non-inflammatory glial properties associated with vascular fluid flow and waste clearance have important roles in disease pathogenesis. Collectively, an emerging body of recent literature highlights the critical role of microglia and astrocytes in the etiology of ALS and FTD.

## Funding

ARC Discovery Grants (DP120100180, DP140103233), NHMRC Project Grant (APP1034816), The Snow Foundation and BitFury.

## Conflict of Interest Statement

The authors declare that the research was conducted in the absence of any commercial or financial relationships that could be construed as a potential conflict of interest.

## Abbreviations

ALS: Amyotrophic lateral sclerosis
Aβ: Amyloid beta
AQP4: Aquaporin 4
CNS: Central Nervous System
CSF: Cerebrospinal Fluid
FTD: Frontotemporal dementia
FUS: Fused in Sarcoma
GRN: Progranulin
iPSC: Inducible Pluripotent Stem Cell
MRI: Magnetic Resonance Imaging
MN: Motor Neuron
NF, κB: Nuclear Factor-kappa B
OPTN: Optineurin
PET: Positron Emission Tomography
PFN1: Profilin 1
RNAi: RNA interference
SOD1: Superoxide Dismutase 1
SPECT: Single Positron Emission Computed Tomography
SQTSM1: Sequestosome 1
Tau: Microtubule Associated Protein Tau
TBK1: Tank Binding Kinase 1
TREM2: Triggering Receptor Expressed on Myeloid cells 2
TDP: 43-TAR DNA-binding 43
VCP: Valosin-Containing Protein.
